# The media morphosis of science communication during crises

**DOI:** 10.1038/s41598-025-88973-7

**Published:** 2025-02-14

**Authors:** Rocco Caferra, Giuseppe Di Liddo, Andrea Morone, David Stadelmann

**Affiliations:** 1 University of Rome Unitelma Sapienza, Rome, Italy; 2 University of Bari “Aldo Moro”, Bari, Italy; 3 University of Bayreuth, Bayreuth, Germany

**Keywords:** Trust, Crises, Media, Knowledge, Public health, Quality of life, Human behaviour

## Abstract

Effective science communication is crucial in informing citizens in times of crisis. The ICT-driven metamorphosis of the media industry (media-morphosis) has facilitated the shift from a rather one-directional information deficit model in science communication to a more bidirectional communication corresponding to a dialogue model. Little is known about the preferences for social media communication from the supply side of institutional and scholarly communication. Analyzing survey data from nearly 8,700 scientists, we compared their preferences for press media and social media, proxies of traditional centralized communication channels and modern decentralized channels, respectively. Our empirical results show that trust in governments and prioritizing access to all perspectives on the crisis are important factors that influence scientists’ preferences regarding forms of communication. Trust favors centralized systems, while social media serves as an alternative in contexts lacking trust in politics. Prioritizing the diffusion of different perspectives drives a shift to stated preferences for decentralized communication systems. We also show that scientists’ stated preferences for communication systems depend on the socio-political context.

**Supplementary Information:**

The online version contains supplementary material available at 10.1038/s41598-025-88973-7.

## Introduction

The advent of the digital age has paved the way for innovations in the interaction between scientific institutions, scientists and citizens^1,2^. Social media platforms are increasingly taking center stage at the expense of traditional print media, leading to a transformation of science journalism^3^. These platforms, supported by the development of e-government and mobile technologies hold the promise to change science communication, stimulate innovation and rethink the flow of information in the “knowledge society”^4,5^. The media industry is undergoing a significant transformation, commonly referred to as the “media-morphosis”^6^. In this transformation process, it is essential to understand the innovations of scientific information dissemination^5^. The accessibility and type of (science) information published is highly dependent on the media platform used^7^. Given the disruptive innovation experienced by the media industry^8^, it is relevant investigating emerging trends in science communication^9^ and, in particular, understanding scientists’ preferences for traditional and modern communication channels.

The media-morphosis can be analyzed by observing the preferences for two types of systems: traditional centralized media, such as print media, and modern social media. These models have different characteristics including the potential quality, accessibility, and effectiveness of the disseminated information. In fact, as analyzed in this paper, traditional centralized models tend to view science as a one-way knowledge transfer, but this has been criticized for being top-down and paternalistic. Modern decentralized models, on the other hand, favor accessibility and the speed of information flow, but they might make filtering and quality checking processes more complicated due to the multitude and heterogeneity of agents present within them. This relative decentralization marks a shift from “information deficit model” which sees public skepticism because of insufficient knowledge, to a potentially more inclusive “dialogue model” which emphasizes mutual understanding and participatory engagement between scientists and the public^10^.

There is a lack of research on the impact of this media-morphosis on the supply of insights by scientists. We aim to gain a deeper understanding of scientists’ preferences regarding science communication during crises, a showcase where the dissemination of the correct information is vital to foster human coordination.

Understanding the factors that influence scientists’ communication preferences is particularly important in times of crisis, whether natural disasters, public health emergencies or other unforeseen events, the importance of rapid and accurate communication is paramount. The information driven coordination and engagement of human efforts can be critical to effectively managing and mitigating the adverse effects of a crisis^11,12^. Ideally, well-structured crisis communications ensure that all stakeholders including politicians, experts, and citizens have access to the necessary information. In this context, the role of the scientific community is crucial, as scientists have the knowledge and insights needed to inform policymakers and the public at large. Indeed, science communication and policy mutually influence each other, and emerging social media science communication could influence the use of scientific evidence in the policy-making process^9^.

To investigate factors affecting scientists’ communication preferences, we analyzed a survey of scientists with nearly 8,700 responses on items related to more centralized or decentralized means of communication. By analyzing scientists’ responses, we explore their propensity for different channels of information dissemination as a function of scientists’ trust in government, their assessment of knowledge in the scientific community, and the importance they place on the diversity of perspectives, including diverging perspectives such as potential conspiracy theories. Specifically, we compare scientists’ preferences for more centralized, top-down systems, such as traditional print media, which are typically curated and disseminated by professional journalists and editors, with more decentralized systems, such as social media, where content is generated and shared by a diverse range of users.

We investigate two hypotheses related to scientists’ preferences for means of communication during emergencies and provide evidence to support them. Firstly, we hypothesize that scientists who trust the government are more likely to favor centralized communication forms, such as print media, while being less inclined towards decentralized communication forms, like social media. As a second hypothesis we propose that when scientists believe that all perspectives on crises should be considered, their preferences will be reversed, favoring decentralized communication over centralized platforms. Through the analysis of survey data collected during the early period of the COVID-19 pandemic (from May 4 to June 3, 2020), we substantiate both hypotheses.

Our empirical findings reveal that trust in government is positively associated with scientists’ preferences for centralized information dissemination. On the other hand, when scientists emphasize the importance of diverse perspectives, they show a tendency to favor decentralized communication channels like social media. Furthermore, our results uncover an additional influencing factor on scientists’ communication preferences. Scientists who perceive that the scientific community has a clear understanding of the costs and benefits of different courses of action during the crises are more inclined to support centralized communication forms while no effect is found for decentralized communication forms. This indicates that the perception of the scientific community’s knowledge plays a significant role in shaping communication preferences of scientists during crises.

 The remainder of this paper is organized as follows: Sect. "Literature review and theoretical framework" overviews the literature motivating our article, while we discuss the data and empirical strategy in Sect. "Data and empirical strategy". Our main results, robustness tests and refinements are presented in Sect. "Results". Section "Conclusions" offers concluding remarks.

## Literature review and theoretical framework

Science communication plays a vital role in enhancing societal culture by fostering scientific awareness and increasing access to information^7^. However, scientific knowledge, often seen as the epitome of ‘true knowledge,’ also relies on mass media to bridge any potential gap between scientists and society. Although different types of outreach events and educational venues (such as the European Researchers’ Night) play a role in communicating scientific knowledge, mass media offers the advantage of large-scale, continuous dissemination.

Media services have experienced significant innovations in recent times^13,14^. Despite its importance, the relationship between innovation theory and media studies remains underexplored. Media innovation includes both process and product changes, such as the emergence of new platforms and novel types of information. These developments are crucial for the transformation of the media industry^8^. Within this framework, digital social media platforms have emerged as disruptive innovations in the internet era, representing a media-morphosis responsible for significant sociopolitical changes^6,15–17^. The shift in the use of media can be analyzed by comparing centralized and decentralized systems of information dissemination. In the following section, we will explain the reasons behind this shift and shed light on why such analyses become particularly relevant in times of crisis.

Traditional print media, operating within centralized, top-down systems, relied on professional journalists and editors to curate content. By contrast, contemporary internet-based models adopt a decentralized, bottom-up approach that fundamentally alters the dissemination dynamics. While traditional media ensures a certain editorial rigor, social media platforms, driven by a diverse range of independent users, can amplify the risk of misinformation and fake news^18,19^.

Social media platforms are often seen as enhancing democracy through open and equal deliberation among citizens, representatives, and policymakers^20^. On the one hand, they facilitate social networking and user-centered innovation^21,22^, enabling the open-source diffusion of information. On the other hand, they risk degrading the quality of information compared to traditional, authoritative mass media. The democratization related to social media may introduce challenges such as misinformation, power imbalances, and cultural impacts^23,24^. This duality highlights the need for improved communication strategies that address these evolving challenges.

Over recent decades and with the advent of social media in particular, science communication has also transitioned from a “information deficit model” to the more participatory approach of a “dialogue model”. This transition has coincided with changes in the creation and dissemination of informational content. In fact, the information deficit model assumes a one-way transfer of knowledge from informed experts to a passive, uninformed audience. The audience functions as a receiver of information. Centralized systems tend to correspond to deficit model because they limit possible interactions between the expert and the audience. This approach has been critiqued for oversimplifying the complex relationship between scientific knowledge and society^10,25^. The dialogue model, characterized by reciprocal engagement and mutual understanding between scientists and the public^26^has been argued to promote active engagement with non-expert communities, emphasizing trust-building rather than mere dissemination^27^. This may foster inclusive and socially robust solutions, further enhancing the societal relevance and impact of research^28^, potentially at the expense of reliability and accuracy. These models find their potential empirical correspondence in in decentralized systems, such as social media, where users receive information and have the opportunity to interact both among themselves and with the information provider. While our empirical analyses relate the dialogue model to social media, it is important to note that it is not limited to social media; rather, it includes all dialogic approaches aimed at fostering a two-way conversation, enabling the exchange of information, perspectives, perceptions, and concerns between scientists and the public^27^.

During crises, the intersection of science and media becomes central to coordinating individual responses and making informed decisions^29–32^. Preferences for communication models, whether centralized (in line with the information deficit model) or decentralized (in line with the dialog model), might depend on trust^33^and the perceived value of diverse perspectives. The limitations of centralized gatekeeping, as highlighted during the early COVID-19 pandemic, underscore the importance of open systems for addressing public confusion and misinformation^34–36^. The expertise of scientists and their communication strategies play a pivotal role in mitigating these issues.

The spread of misinformation during crises^37,38^ underscores the need for robust communication systems. As discussed above, traditional media’s centralized filtering often provides higher-quality information, while decentralized networks promote crowdsourced knowledge influenced by informal networks^39^. Social media platforms facilitate rapid access to and dissemination of information, enabling greater diversity of perspectives^40,41^. However, as Melki et al.^42^ argue, information verification practices are critical, given the questionable credibility of user-generated content. Furthermore, a broader participation in the information diffusion, as in the dialogue model, may improve the effectiveness of communication. These contrasting systems shape communication preferences, particularly during crises when rapid, accurate information is essential.

## Data and empirical strategy

### Data

To comprehensively assess the range of perspectives of scientists regarding centralized and decentralized forms of communication, we explore a survey designed during the period of late March to April 2020, i.e., at the start of the COVID-19 pandemic. At that time, the global community was grappling with the uncertainties associated with the disease and the intricate nuances of knowledge present in the scientific community. The data collection phase spanned from May 4 to June 3, 2020, a period that coincided with the initial wave of COVID-19 in many countries, during which the world’s attention was focused on contrasting the pandemic. The survey allows us to gather unique insights that may inform future discussions during crises when expert knowledge is required.

### Ethical clearance and survey information 

Ethical clearance and protocol approval for data collection was granted by the Ethics Commission of the Frankfurt School of Finance and Management. We follow the legal duty of General Data Protection Regulation (GDPR) (EU) 2016/679. No personal or sensitive information that can be used to identify the respondents were collected. Besides, the consent of the respondents to partake in the online survey were seek before the survey was executed by including an electronic informed consent in the online survey form. All procedures were performed in accordance with relevant guidelines. The participants were self-selected and informed that the survey pertained to the crisis related to the pandemic. As an incentive, participants were offered a $500 lottery prize for themselves and $500 for a charity of their choice if they provided a contact email address at the very end of the survey.

The survey was conducted through SurveyMonkey in 2020 (surveymonkey.com). SurveyMonkey is one of the world’s most popular survey platforms. It targeted scientists who had been corresponding authors of articles published in top-ranked journals across 55 scientific fields from 2015 to early 2020. The selection of journals was based on their 2019 SCImago Journal Ranking (SJR) in 13 scientific areas defined by Scopus: Arts and Humanities; Business, Management and Accounting; Economics, Econometrics and Finance; Energy; Health Professions; Immunology and Microbiology; Medicine; Multidisciplinary; Neuroscience; Nursing; Pharmacology, Toxicology and Pharmaceutics; Psychology; Social Sciences. To obtain responses from corresponding authors, 849 journals were sampled, and 318,251 corresponding author email addresses were extracted from all over the world^43^. To ensure representation of social scientists, we also included 68,470 scholars registered in the RePEc bibliographic database. Two-thirds of the sample pool, amounting to 220,923 invitations, were randomly sent out from May 4 to June 3, 2020, excluding Sundays. The response rate based on opened emails was 13.93%, 5.76% based on accepted invitations, with 98% completing the survey within 24 h of opening the link. The survey covered various topics related to COVID-19, including a special section on the media. Participation was voluntary, and subjects could skip questions or quit the survey at any time. Consequently, there is a proportion of missing values for different variables of about 33%.

### Main variables of interest

Our primary objective is to investigate preferences for centralized and decentralized information channels, particularly focusing on print media vs. social media. To capture scientists’ communication preferences, we utilized a question where participants were asked to indicate the extent to which they obtained information from print media and social media on a 7-point Likert scale, ranging from 1 (not at all) to 7 (very much). The specific question was “*To what extent do you get your information about the COVID-19 epidemic from the following sources?* [item 1 – print media], [item 2 – social media]”. It is worth noting that the two items are negatively correlated, with a correlation coefficient of −0.205 (*p* < .01). On the one hand, there might be some key variables determining the trade-off between the two means of information; on the other hand, the absence of a close inverse correlation can be partially attributed to other determinants explaining the preferences for both the means of communications. We aim to explore these determinants.

A central aspect of our research is investigating how trust in government influences preferences of means of communication. To measure trust in government’s truthfulness and care for citizens, we closely follow the literature and adopt the approach used by Fetzer et al. (2020) and explicitly asked participating scientists whether they trust their country’s government to take care of its citizens (“*How much do you trust the government of the country where you live in to take care of its citizens?*”). Thereby we ensure the quality and comparability of the results with our trust measure^43^ (Forrester and Nowrasteh, 2023). As in the literature, our measure ranges from 1 to 5 Likert scale with higher values reflecting higher levels of trust in governments and, in turn, greater perceived truthfulness of government.

Furthermore, we delve into the direct connection between the media and information dissemination. To explore the hypothesis that scientists favor decentralized communication when they believe in considering all perspectives on a crisis, i.e., including those perspectives deemed as a potential conspiracy, we incorporated a specific question in the survey. Participants were asked to rate their agreement level on a 7-point Likert scale, ranging from 1 (strongly disagree) to 7 (strongly agree) regarding whether the news media should report all perspectives on COVID-19 even in relation to conspiracy (“*The news media should report all perspectives on COVID-19*,* even those considered to be conspiracy theories*.”).

In addition, we aim to understand how scientists’ perceptions of the scientific community’s knowledge influence their communication preferences for centralized vs. decentralized means of communication. To measure this, we asked participants to rate their perceptions of whether the scientific community has a clear understanding of the costs and benefits of different courses of action regarding the coronavirus (“*The scientific community has a clear idea about the costs and benefits of different courses of action in relation to the coronavirus.*”). The responses were recorded on a 7-point Likert scale, ranging from 1 (strongly disagree) to 7 (strongly agree).

In Table 1, we present descriptive statistics for the main variables that provide an overview of the responses received from the participating scientists. It is evident that preferences for decentralized information systems are, on average, higher than those for Centralized ones (4.96 vs. 3.9), i.e. during crisis the overall support for a communication approach in line with the dialogue model is somewhat higher than support for an approach following the deficit model. In addition to these variables, we also control for a wide range of personal characteristics, views on the goals of government policy during the crisis, fixed effects for countries where scientists live, fixed effects for scientific fields, and other relevant covariates, including COVID19 case numbers, among others. These additional variables can help us to better isolate the relationships of interest and the potential influences of trust on scientists’ communication preferences during a critical point of the crisis. Descriptive statistics of all other control variables are relegated to the online supporting information, Table A1.

**Table 1:** Descriptive statistics (Likert Scales).

Variable	Obs.	Mean	Std. Dev.	Min	Max
**CentralizedInfo**: Preference for Centralized Information - To what extent do you get your information about the COVID-19 epidemic from the following sources? (Print media)	8493	3.901	2.314	1	7
**DecentralizedInfo**: Preference for Decentralized Information - To what extent do you get your information about the COVID-19 epidemic from the following sources? (Social Media)	8701	4.96	1.965	1	7
**Trust**: How much do you trust the government of the country where you live in to take care of its citizens?	8509	2.939	1.395	1	5
**AllPerspectives**: The news media should report all perspectives on COVID-19, even those considered to be conspiracy theories.	8438	2.823	1.8	1	7
**KnowledgeCommunity**: The scientific community has a clear idea about the costs and benefits of different courses of action in relation to the coronavirus.	8497	3.968	1.573	1	7

*Notes*: Descriptive statistics for main variables of interest based on responses of individual scientists. Descriptive statistics for all variables are presented in the supporting information.

### Empirical strategy

Our empirical strategy is a regression control framework. Our first main estimation Eq. (1) focusses on preferences for centralized information provision as a dependent variable:

1 $$\:CentralizedInf{o}_{i}=\alpha\:+{\beta\:}_{1}Trus{t}_{i}+{\beta\:}_{2}AllPerspective{s}_{i}+\:{\beta\:}_{3}KnowledgeCommunit{y}_{i}+{\varvec{X}}_{\varvec{i}}\varvec{\beta\:}+{\varvec{C}}_{\varvec{i}}\phi\:+\epsilon\:$$

Our second main estimation Eq. (2) focusses on preferences for decentralized information provision.

2 $$\:DecentralizedInf{o}_{i}=\gamma\:+{\delta\:}_{1}Trus{t}_{i}+{\delta\:}_{2}AllPerspective{s}_{i}+{\delta\:}_{3}KnowledgeCommunit{y}_{i}+{\varvec{X}}_{\varvec{i}}\varvec{\delta\:}+{\varvec{C}}_{\varvec{i}}\gamma\:+\epsilon\:$$

The unit of observation in both equations is the individual scientist. We always control for all personal characteristics, scientific domains, and other covariates summarized by $\:{\varvec{X}}_{\varvec{i}}$. We also account for country-fixed effects, $\:{\varvec{C}}_{\varvec{i}}$. The inclusion of country-fixed effects is able account for potential for country-level heterogeneity, such as cultural differences or the relevance of country-specific institutions. Media usage may differ significantly depending on national or cultural backgrounds. Similarly trust in government may depend on institutions. By accounting for country-fixed effects, we hold the influence of country-specific factors constant. $\:\epsilon\:$ is an idiosyncratic error term. Results are based on OLS estimators, and we calculate robust standard error estimates.

Both estimation equations differ only regarding the dependent variable. Our hypothesis is that scientists who exhibit higher trust levels (*Trust*) tend to favor centralized information provision (*CentralizedInfo*) more than decentralized information provision (*DecentralizedInfo*), i.e. $\:{\beta\:}_{1}>{\delta\:}_{1}$ (Hypothesis 1). It might even be the case that higher trust levels are positively associated to centralized information provision but negatively associated to decentralized information provision, i.e. $\:{\beta\:}_{1}>0$ and $\:{\delta\:}_{1}<0$.

Moreover, we hypothesize that scientists having higher preferences for reporting all perspectives on the crisis (*AllPerspectives*), have a lower tendency to support centralized information provision but a higher tendency to support decentralized information provision, such that $\:{\beta\:}_{2}<{\delta\:}_{2}$ (Hypothesis 2). It might even be the case that scientists who care for all perspectives do not favor centralized information provision, i.e., $\:{\beta\:}_{2}<0$, but favor instead decentralized information provision, i.e., $\:{\delta\:}_{2}>0$.

Finally, if scientists think that the scientific community has a clear idea about the costs and benefits related to the crisis (*KnowledgeCommunity*), then we hypothesize that they rather support a centralized information provision in comparison to a decentralized information provision, i.e., $\:{\beta\:}_{3}>{\delta\:}_{3}$ (Hypothesis 3). We suppose that if scientists think that their community has a comparatively low knowledge of the costs and benefits related to the crisis, then they do not express any clear preference for either centralized or decentralized information provision.

In our empirical analysis, we account for numerous other covariates ($\:{\varvec{X}}_{\varvec{i}}$) to reduce potential omitted variable bias in our coefficient estimates. For example, if older scientists tend to prefer more centralized information provision and if they also have more trust in government, controlling for the age of the scientists responding to our survey is relevant. The large array of covariates and country effects in our analysis gives us some confidence that the relationships found between trust, preference for all perspectives and the belief regarding knowledge in the scientific community may shape preferences for different communication channels. Importantly, we hypothesized that trust in government, preferences for all perspectives to be reported, and the belief regarding knowledge in the scientific community systematically and differentially correlate with preferences for centralized and decentralized information.

Thus, our setting gains the reliability because for precisely the same individuals, the same control variables, the same estimation strategy, we predict that $\:{\beta\:}_{1}>{\delta\:}_{1}$, $\:{\beta\:}_{2}<{\delta\:}_{2}$, and $\:{\beta\:}_{3}>\:{\delta\:}_{3}$, i.e., we predict the differential influences for our main variables regarding centralized versus decentralized information provision. Our results will provide empirical support for all three hypotheses.

## Results

### Main results

Table 2 presents the key findings of our study. In specifications (1) to (4), we examine scientists’ preferences for centralized information as a dependent variable, while in specifications (5) to (8), we investigate preferences for decentralized information provision as a dependent variable. To estimate specifications (4) and (8), we make use of ordered probit models. To ensure the robustness of our results and account for potential confounding factors, we always incorporate a comprehensive set of control variables across all specifications, along with country effects. Coefficients for all control variables are presented in the supporting information.

If scientists express trust in their country’s government to take care of citizens, they generally show a preference for centralized information provision, such as print media. The estimated point coefficient associated with the variable *Trust* exhibits a positive and statistically significant association, $\:{\beta\:}_{1}>0$, in all specifications where the dependent variable is preferences for centralized information. On the other hand, higher levels of trust in government are negatively associated ($\:{\delta\:}_{1}<0$) with preferences for decentralized information provision, such as social media. This association is, however, not statistically significant. Consequently, our findings support Hypothesis 1 that the coefficient for preferences of centralized information is greater than the coefficient for preferences of decentralized information, $\:{\beta\:}_{1}>{\delta\:}_{1}$. Scientists who exhibit higher trust levels tend to favor centralized information provision.

Next, we delve into the association between the support expressed by scientists for news media reporting all perspectives, including those considered as conspiracy theories. The variable representing this support, *AllPerspectives* is found to be negatively and statistically significantly associated with preferences for centralized information provision, $\:{\beta\:}_{2}<0$. In contrast, when considering decentralized information provision as the dependent variable, the coefficient for *AllPerspectives* is positively and statistically significantly associated with this variable, $\:{\delta\:}_{2}>0$. This implies that scientists who express a stronger preference for news media reporting all perspectives on the crisis are less likely to support centralized information provision but are more inclined to support decentralized information provision. In essence, their willingness to consider diverse perspectives appears to influence their communication preferences, favoring decentralized channels over centralized ones. Thereby providing support for Hypothesis 2, i.e., $\:{\beta\:}_{2}<{\delta\:}_{2}$.

**Table 2:** Differential influence of trust and preferences for reporting all perspectives in the media on . Centralized vs. decentralized information provision.

	Centralized Information				Decentralized Information			
	(1)	(2)	(3)	(4 ordered probit)	(5)	(6)	(7)	(8 ordered probit)
Trust	0.079***		0.077***	0.036***	− 0.031		− 0.026	− 0.014
	(0.023)		(0.024)	(0.012)	(0.02)		(0.02)	(0.011)
AllPerspectives		− 0.033**	− 0.032**	− 0.014*		0.086***	0.084***	0.046***
		(0.016)	(0.016)	(0.008)		(0.014)	(0.014)	(0.008)
KnowledgeCommunity	0.088***	0.081***	0.078***	0.039***	0.019	0.021	0.022	0.014
	(0.018)	(0.018)	(0.018)	(0.009)	(0.015)	(0.015)	(0.015)	(0.009)
Control Variables	yes	yes	yes	yes	yes	yes	yes	yes
Country effects	yes	yes	yes	yes	yes	yes	yes	yes
Observations	7535	7394	7379	7379	7707	7556	7540	7540
(Pseudo) R2	0.12	0.121	0.122	0.036	0.079	0.083	0.083	0.027

*Notes*: Robust standard errors are in parentheses; *** *p* < .01, ** *p* < .05, * *p* < .1.

Finally, we explore the significance of scientists’ beliefs regarding the knowledge of the scientific community concerning the costs and benefits of various courses of action related to the crisis. Our results indicate that as the perceived knowledge of the scientific community increases, scientists’ preferences for centralized information also increase significantly. However, we find no statistically significant relationship between the variable *KnowledgeCommunity* and preferences for decentralized information provision. Providing evidence for Hypothesis 3, our findings suggest that the higher the level of perceived knowledge within the scientific community, the more likely an expert is to support centralized information provision over decentralized information provision, $\:{\beta\:}_{3}>{\delta\:}_{3}$. This underscores the relevance of expert perceptions of the scientific community’s expertise in shaping their communication preferences during the crisis.

These results highlight that individuals who have higher trust in government are more likely to prefer top-down systems, supporting information providing in line with the deficit model during times of crisis, where one-directional information diffusion between experts and non-experts is the standard. Still, it is important to note that preferences for dialogue model, based on the use of decentralized social media networks, are generally higher, supporting the earlier argument about the social transition toward this type of system (as highlighted in Table 1). Moreover, these preferences are neutral with respect to institutional trust (the impact is not statistically significant), do not depend on the perception of the quality of information held by experts, but rather follow a bottom-up model where scientists support the democratization and heterogeneity of the information being disseminated.

### Robustness and refinements: the relevance of social trust and quality of government

We further explore our main hypotheses concerning the relevance of scientists’ trust and preferences for reporting all perspectives in the media. Considering the model outlined in the empirical strategy section, we made several extensions using the sample splitting approach. To provide a deeper analysis, we considered two additional dimensions: social trust in society and the quality of government, since formal and informal institutions can play an important role in defining social organization^44,45^. To achieve this, we divided the sample of scientists based on the countries in which they are active. Specifically, we formed groups of scientists from countries with low levels of social trust and high levels of social trust, as well as groups from countries with low government quality and high government quality. This setting allows us to investigate the relevance of institutions (including social trust in a country as well as government quality) next to individual responses of scientists. We then estimate our main models including all control variables and country effects. The results of this analysis are presented in Table 3.

We have observed that the individual trust of scientists in the government’s ability to take care of its citizens during the crisis is consistently positively associated with preferences for centralized information provision. This association holds true regardless of whether the country has a low trust or high trust society and irrespective of the quality of government as show in Table 3. Thus, the coefficient $\:{\beta\:}_{1}$ for *Trust* remains positive regardless of the specific institutional context, indicating a consistent pattern of trust positively influencing preferences for centralized information provision. Conversely, we find that trust is never positively associated with preferences for decentralized information provision. In certain settings where there is high social trust and high government quality, there is even a slightly negative association ($\:{\delta\:}_{1}<0$). Thus, our main hypothesis regarding the relevance of trust in government among scientists remains robust. Across different contexts, we systematically observe that $\:{\beta\:}_{1}>{\delta\:}_{1}$ (Hypothesis 1) indicating that scientists with higher levels of trust in government tend to prefer centralized information provision over decentralized alternatives, favoring the one-way communication of the information deficit model.

**Table 3:** The relevance of social trust and government quality for the differential influence of individual trust and preferences for reporting all perspectives in the media.

	Centralized Information		Decentralized Information		Centralized Information		Decentralized Information	
	(1) Low Social trust	(2) High Social trust	(3) Low Social trust	(4) High Social Trust	(5) Low Government Quality	(6) High Government Quality	(7) Low Government Quality	(8) High Government Quality
Trust	0.071*	0.078***	0.01	− 0.043*	0.082*	0.075***	0.011	− 0.041*
	(0.042)	(0.029)	(0.033)	(0.025)	(0.044)	(0.029)	(0.035)	(0.025)
AllPerspectives	0.016	− 0.053***	0.096***	0.078***	0.029	− 0.057***	0.072***	0.089***
	(0.028)	(0.02)	(0.023)	(0.018)	(0.029)	(0.02)	(0.023)	(0.017)
KnowledgeCommunity	0.089***	0.07***	0.036	0.017	0.111***	0.063***	0.028	0.021
	(0.034)	(0.021)	(0.027)	(0.019)	(0.035)	(0.021)	(0.028)	(0.018)
Control Variables	yes	yes	Yes	yes	yes	yes	yes	yes
Country effects	yes	yes	Yes	yes	yes	yes	yes	yes
Observations	1982	5397	2036	5504	1817	5562	1870	5670
(Pseudo) R2	0.16	0.113	0.157	0.059	0.184	0.103	0.156	0.057

*Notes*: Robust standard errors are in parentheses; *** *p* < .01, ** *p* < .05, * *p* < .1.

When investigating the variable *AllPerspectives*, a more nuanced picture emerges. As before, we consistently find a positive relationship in all institutional settings with a preference for decentralized information ($\:{\delta\:}_{2}>0$). However, in societies with high social trust and high government quality, scientists who express a higher preference for having all perspectives reported tend to show a lower preference for centralized information provision ($\:{\beta\:}_{2}<0$), supporting participatory in line with the dialogue model. On the other hand, there is no correlation between preferences for all perspectives and centralized information provision in societies with low social trust and low government quality. This observation can be viewed as consistent, as in such societies, there might be a perception of a relevant risk when reporting all perspectives, including conspiracy theories. Our empirical result also highlights the complex interplay between societal factors, trust, and preferences for information dissemination during crises. The role of social trust and government quality adds further depth to our understanding of how scientists’ communication preferences are influenced by diverse perspectives, underscoring the importance of context in shaping their preferences. Overall, our empirical results offer further support for Hypothesis 2. Scientists who favor that all perspectives on a crisis should be reported in the media display a lower tendency to support centralized information provision but exhibit a higher tendency to support decentralized information provision ($\:{\beta\:}_{2}<{\delta\:}_{2}$).

Finally, our analysis considers the influence of scientists’ perceptions regarding the knowledge of the scientific community on costs and benefits related to the crisis depending on social trust and government quality. We find that if scientists believe the scientific community possesses a clear understanding of such aspects, they consistently express support for centralized information provision. This association remains irrespective of whether they are in a low social trust society or a high social trust society, as well as whether they reside in a country with low government quality or high government quality. In essence, the coefficient $\:{\beta\:}_{3}\:$for *KnowledgeCommunity* remains positive and aligned with our hypotheses. On the other hand, we do not observe any significant association between the variable *KnowledgeCommunity* and preferences for decentralized information provision. As such, the perceived knowledge within the scientific community does not seem to impact scientists’ preferences for decentralized information dissemination ($\:{\delta\:}_{3}$). In conclusion, we observe $\:{\beta\:}_{3}>{\delta\:}_{3}$ (consistent with Hypothesis 3) such that the results demonstrate that scientists who have confidence in the scientific community’s understanding of the costs and benefits related to the crisis tend to support centralized information provision consistently across diverse societal contexts. However, their perceptions of knowledge do not seem to play a significant role in shaping their preferences for decentralized information provision.

### Discussion and limitations

We analyze how different models of science communication are perceived and accepted by scientists, particularly during crises when the dissemination of accurate information is vital for social coordination. Our results show that scientists with higher levels of trust in government are more likely to favor centralized information dissemination, consistent with the information deficit model. Conversely, scientists who prioritize the inclusion of all perspectives in media reporting, even those considered conspiratorial, demonstrate a stronger preference for decentralized communication channels, in line with the dialogue model. Finally, when scientists believe that the scientific community has a clear understanding of the costs and benefits related to a crisis, they are more likely to support centralized information provision. This suggests that in situations where scientific consensus exist, the need for feedback and dialogue through decentralized models may be perceived as less critical. Our findings overall suggest a growing preference for decentralized, participatory communication models aligned with the dialogue model. Interestingly, the preference for decentralized models persists even in times of crisis but it is dependent on trust, perceptions regarding knowledge of the scientific community and the relevance assigned to the inclusion of all perspectives in science communication.

While the study encompasses a large sample of scientists globally, making the findings less susceptible to biases stemming from national, cultural, or institutional differences as when the analysis was based on scientists from a single country, it is important to recognize the contextual limitations. Our data were collected during a specific and unique crisis: the peak of the first wave of the COVID-19 pandemic. This context may limit the generalizability of our results to other crises and to how scientists view the role of science communication in general. Nevertheless, studying a crisis scenario offers valuable insights, as such situations often intensify conflicts between centralized and decentralized information provision, providing a critical testbed for understanding scientists’ communication preferences.

Although our analysis accounts for country-fixed effects and a range of control variables, it relies on observational data from a survey. This methodological approach inherently limits the ability to establish causality. Unobservable factors potentially correlated with our main variables of interest and the preference for centralized or decentralized communication forms might affect the coefficient estimates. However, the observed variations in scientists’ preferences, as well as the influence of trust and the perceived importance of including all perspectives, suggest systematic differences in how centralized and decentralized information systems are viewed and which model of science communication matters during crises.

It is also important to acknowledge that we treat all forms of decentralized media, such as social media, as a single category which is a limitation of our study. We do not differentiate between platforms like X, Facebook, YouTube, or blogs, each of which may have distinct user characteristics and attract varying preferences from scientists. Scientists’ attitudes toward these platforms could vary based on factors such as audience reach, content format, or perceived credibility. However, we believe that the stark contrast between centralized and decentralized modes of science communication highlighted in our study remains highly relevant. Our findings suggest that, despite platform-specific nuances, social media in general is perceived as fundamentally different from traditional, centralized media forms. This distinction provides meaningful insights into how decentralized systems are viewed as an alternative to conventional communication channels.

## Conclusions

By analyzing a comprehensive survey of up to 8,700 scientists, we investigate their communication preferences during crises, shedding light on the factors that influence their choices between centralized and decentralized information channels. The findings reveal the significant impact of three key variables: trust in government, consideration of diverse perspectives, and perceptions of scientific community knowledge.

Our first result shows that scientists who trust the government tend to favor information provision by means of traditional print media. This preference for centralized systems emphasizes their belief in government capacity and coordination during crises. In contrast, the second hypothesis highlights that when scientists prioritize the reporting of all perspectives, including those considered as conspiracy theories, their preferences shift towards decentralized information platforms, like social media. This observation indicates a desire for diversity in information sources, allowing for open debates and the exploration of alternative solutions. Moreover, our analysis uncovers the relevance of scientists’ perceptions regarding the knowledge of the scientific community on costs and benefits related to the crisis. When scientists perceive consensus in this area, they show a stronger preference for centralized information provision. This suggests their trust in the expertise of the scientific community to guide coordinated mass action during crises.

Trust in institutions and the consensus of scientific thinking strengthen preferences for the use of traditional media and thus for the centralization of information to coordinate human action during crises. Following the preferences of scientists, innovations in social media become important in stimulating the debate on different alternative theories which might potentially be regarded as a kind of “democratization of science” which itself depend less on the quality of institutions and central information systems.

Developing effective crisis communication strategies requires careful consideration of these factors, fostering open dialogue, and ensuring that diverse perspectives are considered.

Future studies could build on this work, expanding it in several directions. From a methodological standpoint, it would be interesting to distinguish the specific characteristics and diversity of actors within social media platforms. In current systems, both authoritative sources (such as expert journalists in print media) and other types of sources within decentralized networks, like social media, coexist. Moreover, it would be relevant to understand the dynamics within decentralized networks to assess whether dialogue model works better in terms of information quality when expert involvement as ambassadors facilitates the promotion and spread of virtuous behaviors, or whether they are influenced by other actors who may hinder the exchange of high-quality information.

Our study is the first to comprehensively examine scientists’ preferences for centralized vs. decentralized forms of communication. It explores the pivotal roles of trust, the belief that all perspectives should be considered, and the perceived knowledge within the scientific community as key factors influencing preferences for centralized or decentralized communication channels. These findings shed light on the broader relevance of the information deficit model versus the dialogue model in shaping science communication strategies, particularly in contexts requiring effective dissemination and engagement.

## Electronic supplementary material

Below is the link to the electronic supplementary material.

Supplementary Material 1

## Data Availability

The dataset, scripts and the survey questions employed in this study are available as an online resource on https://doi.org/10.6084/m9.figshare.28263500.v1.
