# Supplementary material for: The media morphosis of science communication during crises
Source: Sci Rep. 2025 Feb 14;15:5506. doi: 10.1038/s41598-025-88973-7 (PMC11829029; doi:10.1038/s41598-025-88973-7)
Supplement: Supplementary file 1 — Supplementary Material 1 [file 41598_2025_88973_MOESM1_ESM.docx]

# **Supporting Information for Paper: “The Media Morphosis of Science Communication during Crises”**

In what follows, we report the description of the main variables employed (Table A1) , and the full estimates with the coefficients of the control variables (Table A2) following the same order of Table 2. Specifically, we control for sociodemographic features as *i)* gender, age, political view (from liberal-1 to conservative-7*), ii)* academic field and position, and *iii)* personal opinions about the health, social, and economic priorities in the government interventions. As reported along the paper, we also consider as contextual variables the Government Quality Ranking (Effectiveness, Governance Indicators Data), the Control of Corruption ranking (Governance Indicators Data), and Country Social Trust (World Value Survey Data). We also control for the severity of COVID-19 at country level, including the logarithm of the cases per capita and the stringency index from the <https://ourworldindata.org/> platform.

| **Descriptive Statistics** |  |  |  |  |  |
| --- | --- | --- | --- | --- | --- |
| **Variable** | **Obs** | **Mean** | **Std. Dev.** | **Min** | **Max** |
| **DecentralizedInfo:** To what extent do you get your information about the COVID-19 epidemic from the following sources?: Social Media | 8701 | 4.96 | 1.965 | 1 | 7 |
| **CentralizedInfo:** To what extent do you get your information about the COVID-19 epidemic from the following sources?: Print media | 8493 | 3.901 | 2.314 | 1 | 7 |
| **Trust:** Q51. How much do you trust the government of the country where you live in to take care of its citizens? | 8509 | 2.939 | 1.395 | 1 | 5 |
| **AllPerspectives:** Q41. The news media should report all perspectives on COVID-19, even those considered to be conspiracy |  |  |  |  |  |
| theories. | 8438 | 2.823 | 1.8 | 1 | 7 |
| **KnowledgeCommunity:** Q9. The scientific community has a clear idea about the costs and benefits of different courses of action in relation to the coronavirus. | 8497 | 3.968 | 1.573 | 1 | 7 |
| Q19a. The government of the country where I live should focus on Saving as many lives as possible | 8670 | 5.78 | 1.458 | 1 | 7 |
| Q19c. The government of the country where I live should focus on The societal consequences of the coronavirus | 8663 | 5.627 | 1.281 | 1 | 7 |
| Q19d. The government of the country where I live should focus on the Economic stability | 8662 | 5.095 | 1.395 | 1 | 7 |
| **Socio-demographics** | . | . | . | . | . |
| Gender: Male | 8693 | .59 | .492 | 0 | 1 |
| Gender: Female | 8693 | .403 | .49 | 0 | 1 |
| Gender: Other | 8693 | .002 | .04 | 0 | 1 |
| Gender: Prefer not to say | 8693 | .006 | .076 | 0 | 1 |
| Political View | 8185 | 2.785 | 1.353 | 1 | 7 |
| Age group | 8630 | 3.14 | 1.266 | 1 | 6 |
| **Science Domain** | . | . | . | . | . |
| Applied Sciences | 8626 | .065 | .247 | 0 | 1 |
| Arts & Humanities | 8626 | .023 | .149 | 0 | 1 |
| Economic & Social sciences | 8626 | .383 | .486 | 0 | 1 |
| Health Sciences | 8626 | .394 | .489 | 0 | 1 |
| Natural Sciences | 8626 | .136 | .343 | 0 | 1 |
| **Position** |  |  |  |  |  |
| Research Associate, PhD Student, Lecturer (or equivalent) | 8665 | 0.170 | 0.376 | 0 | 1 |
| Instructor (or equivalent) | 8665 | 0.037 | 0.190 | 0 | 1 |
| Senior Instructor (or equivalent) | 8665 | 0.030 | 0.173 | 0 | 1 |
| Master Instructor (or equivalent) | 8665 | 0.015 | 0.124 | 0 | 1 |
| Position in the private sector | 8665 | 0.024 | 0.154 | 0 | 1 |
| Other (please specify) | 8665 | 0.032 | 0.176 | 0 | 1 |
| Post-Doctoral Position | 8665 | 0.032 | 0.177 | 0 | 1 |
| Assistant Professor (or equivalent) | 8665 | 0.164 | 0.370 | 0 | 1 |
| Associate Professor (or equivalent) | 8665 | 0.179 | 0.383 | 0 | 1 |
| Professor (or equivalent) | 8665 | 0.228 | 0.419 | 0 | 1 |
| Distinguished, Endowed or University Professor | 8665 | 0.059 | 0.236 | 0 | 1 |
| **COVID-19 Dynamics** |  |  |  |  |  |
| Stringency index | 8663 | 71.492 | 8.607 | 18.52 | 100 |
| ln case per capita | 8608 | 7.545 | 1.293 | 0 | 9.11 |
| **Context Variable** |  |  |  |  |  |
| Government Quality Ranking (Effectiveness, Governance Indicators Data) | 8628 | 81.845 | 16.598 | 1.923 | 100 |
| Country Social Trust (World Value Survey Data) | 8198 | 37.45 | 14.195 | 2.14 | 73.73 |
| Control of Corruption ranking (Governance Indicators Data) | 8628 | 80.02 | 18.252 | .481 | 100 |

**Table A 1** Descriptive Statistics.

|  | **Centralized Information** | | | | **Decentralized Information** | | | |
| --- | --- | --- | --- | --- | --- | --- | --- | --- |
|  | **(1)** | **(2)** | **(3)** | **(4)** | **(5)** | **(6)** | **(7)** | **(8)** |
| **Trust** | .079*** |  | .077*** | .036*** | -.031 |  | -.026 | -.014 |
|  | (.023) |  | (.024) | (.012) | (.02) |  | (.02) | (.011) |
| **AllPerspectives** |  | -.033** | -.032** | -.014* |  | .086*** | .084*** | .046*** |
|  |  | (.016) | (.016) | (.008) |  | (.014) | (.014) | (.008) |
| **KnowledgeCommunity** | .088*** | .081*** | .078*** | .039*** | .019 | .021 | .022 | .014 |
|  | (.018) | (.018) | (.018) | (.009) | (.015) | (.015) | (.015) | (.009) |
| Q19a. The government of the country where I live should focus on Saving as many lives as possible | .007 | .011 | .007 | .01 | .043** | .043** | .045** | .029*** |
|  | (.019) | (.02) | (.02) | (.01) | (.017) | (.018) | (.018) | (.01) |
| Q19c. The government of the country where I live should focus on The societal consequences of the coronavirus | .039 | .041* | .044* | .022* | -.01 | -.014 | -.013 | -.002 |
|  | (.024) | (.024) | (.024) | (.012) | (.021) | (.021) | (.022) | (.012) |
| Q19d. The government of the country where I live should focus on the Economic stability | .02 | .026 | .019 | .011 | .075*** | .072*** | .072*** | .048*** |
|  | (.022) | (.022) | (.022) | (.011) | (.019) | (.019) | (.019) | (.011) |
| **Position** |  |  |  |  |  |  |  |  |
|  |  |  |  |  |  |  |  |  |
| Research Associate, PhD Student, Lecturer (or equivalent) | -.109 | -.109 | -.096 | -.028 | .097 | .11 | .106 | .092 |
|  | (.172) | (.172) | (.173) | (.083) | (.144) | (.145) | (.146) | (.079) |
| Instructor (or equivalent) | .042 | .037 | .056 | .067 | -.048 | -.019 | -.026 | .012 |
|  | (.204) | (.205) | (.206) | (.098) | (.176) | (.176) | (.177) | (.096) |
| Senior Instructor (or equivalent) | -.039 | -.054 | -.043 | 0 | .089 | .111 | .107 | .071 |
|  | (.216) | (.216) | (.218) | (.104) | (.182) | (.182) | (.183) | (.1) |
| Master Instructor (or equivalent) | -.026 | -.034 | -.021 | -.013 | -.093 | -.041 | -.045 | .001 |
|  | (.259) | (.262) | (.263) | (.125) | (.223) | (.226) | (.226) | (.12) |
| Post-Doctoral Position | -.167 | -.163 | -.144 | -.053 | .205 | .225 | .219 | .159 |
|  | (.218) | (.219) | (.22) | (.107) | (.179) | (.179) | (.18) | (.099) |
| Assistant Professor (or equivalent) | .11 | .129 | .143 | .1 | .171 | .175 | .171 | .128 |
|  | (.174) | (.174) | (.176) | (.084) | (.145) | (.146) | (.147) | (.08) |
| Associate Professor (or equivalent) | .14 | .136 | .157 | .106 | .162 | .184 | .178 | .133* |
|  | (.171) | (.172) | (.173) | (.083) | (.143) | (.143) | (.144) | (.078) |
| Professor (or equivalent) | .219 | .2 | .228 | .148* | .104 | .125 | .113 | .088 |
|  | (.171) | (.171) | (.172) | (.082) | (.144) | (.144) | (.145) | (.078) |
| Distinguished, Endowed or University Professor | .404** | .353* | .397** | .227** | .092 | .123 | .121 | .09 |
|  | (.192) | (.193) | (.194) | (.093) | (.17) | (.17) | (.172) | (.091) |
| Position in Private Sector | -.234 | -.247 | -.239 | -.086 | .235 | .21 | .218 | .151 |
|  | (.235) | (.236) | (.238) | (.114) | (.197) | (.198) | (.199) | (.11) |
| Other (please specify) | .095 | .144 | .156 | .101 | .248 | .282 | .275 | .201** |
|  | (.21) | (.213) | (.214) | (.103) | (.181) | (.182) | (.183) | (.102) |
| **Gender** |  |  |  |  |  |  |  |  |
|  |  |  |  |  |  |  |  |  |
| Female | .161*** | .142** | .151*** | .075*** | .024 | .014 | .013 | .016 |
|  | (.056) | (.056) | (.056) | (.028) | (.048) | (.048) | (.048) | (.027) |
| Other | .173 | .141 | .178 | -.008 | .185 | .156 | .146 | .104 |
|  | (.598) | (.593) | (.597) | (.323) | (.536) | (.544) | (.544) | (.322) |
| Prefer not to say | -.219 | -.216 | -.21 | -.083 | .27 | .204 | .203 | .123 |
|  | (.377) | (.377) | (.374) | (.18) | (.301) | (.325) | (.323) | (.184) |
| Political views (liberal-conservative) | -.125*** | -.107*** | -.113*** | -.056*** | -.036** | -.064*** | -.062*** | -.038*** |
|  | (.021) | (.022) | (.022) | (.011) | (.018) | (.019) | (.019) | (.011) |
| Age | .37*** | .382*** | .379*** | .183*** | -.2*** | -.213*** | -.212*** | -.113*** |
| **COVID-19 Dynamics** |  |  |  |  |  |  |  |  |
|  |  |  |  |  |  |  |  |  |
|  | (.026) | (.027) | (.027) | (.013) | (.024) | (.024) | (.024) | (.013) |
| Stringency Index | -.001 | -.001 | 0 | -.001 | .016*** | .014** | .014** | .008** |
|  | (.007) | (.007) | (.007) | (.004) | (.006) | (.006) | (.006) | (.004) |
| lncasepercap | -.036 | .004 | .001 | .01 | -.243 | -.261* | -.262* | -.131 |
|  | (.175) | (.176) | (.176) | (.087) | (.149) | (.149) | (.15) | (.087) |
| **Science Domain** |  |  |  |  |  |  |  |  |
|  |  |  |  |  |  |  |  |  |
| Arts & Humanities | .272 | .288 | .287 | .119 | .13 | .14 | .14 | .071 |
|  | (.199) | (.201) | (.201) | (.098) | (.159) | (.159) | (.159) | (.094) |
| Economic & Social Sciences | .49*** | .453*** | .463*** | .235*** | .026 | .031 | .027 | .01 |
|  | (.11) | (.111) | (.111) | (.056) | (.093) | (.093) | (.093) | (.055) |
| Health Sciences | -.004 | -.038 | -.033 | -.008 | -.417*** | -.398*** | -.4*** | -.244*** |
|  | (.11) | (.111) | (.111) | (.055) | (.094) | (.094) | (.094) | (.055) |
| Natural Sciences | .169 | .137 | .148 | .076 | -.191* | -.185* | -.184* | -.116* |
|  | (.124) | (.125) | (.125) | (.062) | (.105) | (.105) | (.105) | (.061) |
| **Country Effects** | **yes** | yes | yes | yes | yes | yes | yes | yes |
|  |  |  |  |  |  |  |  |  |
| Observations | 7535 | 7394 | 7379 | 7379 | 7707 | 7556 | 7540 | 7540 |
| Pseudo R2 | 0.12 | 0.121 | 0.122 | .036 | 0.079 | 0.083 | 0.083 | .027 |
|  |  |  |  |  |  |  |  |  |

**Table A 2** Full estimates.
